# Joint effects of elevated homocysteine levels and low eGFR on post-stroke cognitive impairment

**DOI:** 10.3389/fneur.2025.1611140

**Published:** 2025-08-04

**Authors:** Chunyan Zhang, Xueqin Cao, Chen Liu, Pengfei Meng, Huizhong Gao, Bo Bai, Cunshui Xue

**Affiliations:** ^1^Department of Neurology, Second Hospital, Shanxi Medical University, Taiyuan, China; ^2^College of Basic Medical Sciences, Tongji Medical College, Huazhong University of Science and Technology, Wuhan, China

**Keywords:** serum homocysteine, estimated glomerular filtration rate, post-stroke cognitive impairment, acute ischemic stroke, adult

## Abstract

**Introduction:**

The correlation between serum homocysteine levels and post-stroke cognitive impairment (PSCI) remains inconsistent. This study aimed to investigate whether serum homocysteine levels are independently associated with PSCI and to assess the effects of renal function on this relationship.

**Methods:**

A retrospective analysis was conducted in 608 patients with ischemic stroke. Homocysteine levels were obtained from inpatient medical records, and global cognitive function status 1 month after discharge was assessed using the Mini-Mental State Examination (MMSE) and the Montreal Cognitive Assessment (MoCA). The relationship between homocysteine levels and PSCI was evaluated using univariate and multiple linear and logistic regression analyses.

**Results:**

The mean age of the patients was 66.6 ± 4.1 years, with 48% being female. The median homocysteine level was 13.8 μmol/L (interquartile range [IQR], 11.3–17.3 μmol/L), and 39.3% of patients had total homocysteine levels above the cutoff of 15 μmol/L. After full adjustment, a stronger positive association between homocysteine levels and PSCI was observed in patients with low estimated glomerular filtration rate (eGFR), with significant interactions between eGFR and MMSE scores (*P* for interaction = 0.005) and between eGFR and MoCA scores (*P* for interaction = 0.001). Joint analyses indicated that the highest risk of PSCI was in patients with eGFR < 90 ml/min/1.73 m^2^ and homocysteine levels ≥15 μmol/L (odds ratios [ORs] were 2.50 [95% CI: 1.49, 4.18; *p* < 0.001] for MMSE and 13.53 [95% CI: 6.64, 27.56; *p* < 0.001] for MoCA in the fully adjusted model).

**Conclusion:**

These findings highlight the additive value of hyperhomocysteinemia and lower eGFR in predicting incident PSCI risk.

## Introduction

Worldwide, PSCI is major sources of post-stroke morbidity and mortality (1) and is highly prevalent among stroke survivors in China (2). The risk of PSCI is a major concern for patients and their families. Therefore, understanding the mechanisms and modifiable determinants of PSCI is of clinical importance in developing preventive strategies and is key to delivering equitable health care to the Chinese population (3, 4).

Homocysteine is a non-essential sulfur-containing amino acid that is produced in the metabolic cycle by demethylation of methionine. It plays a central role in the methionine and folate cycles, and its metabolism is dependent on folate, vitamins B12 and B6. To date, some studies have suggested that elevated homocysteine levels are modifiable risk factors for Alzheimer’s disease (AD) and vascular dementia (VaD) (5–7). For more than four decades, hyperhomocysteinemia, defined as elevated serum homocysteine levels, has been widely recognized as a risk factor for vascular disease and VaD, supported by extensive clinical evidence (8–15). Recently, an umbrella review found hyperhomocysteinemia to be associated with cognitive impairment (16). An increasing number of reviews have shown that the plasma homocysteine level could be a potential biomarker for PSCI (17–19). A meta-analysis found higher homocysteine levels in individuals with AD and indicated that the homocysteine level can be used as an indicator to differentiate between AD and VaD (9). A new study suggests that high homocysteine levels are associated with the progression from mild cognitive impairment to dementia (20). One study demonstrated a correlation between elevated serum homocysteine levels and PSCI, with the former likely serving as a predictive factor for the latter (21). Another study found that elevated homocysteine levels were independently associated with cognitive impairment in a post-stroke population younger than 65 years (22).

The incidence of chronic kidney disease (CKD) is high among individuals with hyperhomocysteinemia, and hyperhomocysteinemia may be an independent risk factor for CKD as well as cardiovascular complications (23, 24). Recently, a study indicated that homocysteine is independently correlated with cognitive function in patients on maintenance hemodialysis (25). Studies have shown that hyperhomocysteinemia predicts an increased risk of inflammation and endothelial damage, which can lead to cardiovascular disease, stroke, and chronic kidney disease (26). Our prior investigation revealed that a decline in estimated glomerular filtration rate (eGFR) may serve as an effective indicator of cognitive impairment following a stroke (27).

Given the established relationships among hyperhomocysteinemia, chronic kidney disease (CKD), and cognitive function following stroke, it is crucial to investigate the precise interactions between homocysteine levels, kidney function indicators, and PSCI. Furthermore, understanding whether renal function indicators influence the association between homocysteine levels and PSCI is essential for the effective prevention and management of cognitive impairment following stroke. Therefore, this study aimed to investigate whether homocysteine levels are independently associated with PSCI and to assess the effects of renal function on this relationship.

## Materials and methods

### Data source

In this retrospective study, 608 patients aged 60 to 80 years with acute ischemic stroke, admitted within 72 h of stroke onset between January 2016 and December 2020, were analyzed. Data were obtained from hospital records and the outpatient cognitive assessment database, collected 1 month after discharge. Data collection began on September 1, 2019. During or after data collection, investigators had access to information that could identify individual patients. None of the patients had a history of severe cognitive impairment prior to stroke, and all were able to complete the assessment.

The study was conducted at the Department of Neurology, the Second Hospital of Shanxi Medical University, Taiyuan, a tertiary hospital in Shanxi, China. This study was approved by the Ethics Committee of the Second Hospital of Shanxi Medical University (No. 2019YX214) and was completed in accordance with the principles of the Declaration of Helsinki. Given the retrospective nature of the study and the anonymized data analysis, informed consent from the patients was not required.

### Clinical and laboratory data

Clinical information obtained from hospital records included systolic and diastolic blood pressure at admission, self-reported demographic characteristics (age, sex, weight, height, education, smoking and drinking status, and history of hypertension and diabetes mellitus), and laboratory test results (homocysteine, blood lipids, fasting glucose, vitamin B12, folate, and creatinine levels). Fasting venous blood samples were collected on the morning following admission. Biochemical measurements were performed using automatic clinical analyzers (Beckman Coulter) at the core laboratory of the Second Hospital, Shanxi Medical University, Taiyuan, China. Serum homocysteine levels were measured using an enzymatic cycling method, and hyperhomocysteinemia was defined as a homocysteine concentration greater than 15 μmol/L. Serum vitamin B12 and folate levels were measured using a chemiluminescent immunoassay. Serum glucose levels were determined by the hexokinase method. The concentrations of serum creatinine and lipids were measured using enzymatic methods.

In addition, body mass index (BMI) was calculated as weight in kilograms divided by height in meters squared. Neurological function was assessed using the form of the National Institutes of Health Stroke Scale (NIHSS) score (28). The patients with acute ischemic stroke were diagnosed with large artery atherosclerosis, small artery occlusion, cardioembolism, other determined cause, or undetermined cause according to the Trial of Org 10,172 in acute stroke treatment (TOAST) classification (29). The estimated glomerular filtration rate (eGFR) was calculated using the Chronic Kidney Disease Epidemiology Collaboration (CKD-EPI) equation (30).

### Cognitive function assessment

All cognitive function assessments were performed by two trained neuropsychological evaluators using the MMSE and MoCA, 1 month after discharge from the hospital. MMSE and MoCA scores range from 0 to 30, with higher scores indicating better cognitive function. The cognitive domains assessed include concentration, attention, language, orientation, immediate and short-term recall, and the ability to follow simple verbal and written commands. Based on previous studies and the cognitive scores of patients, moderate-to-severe cognitive impairment 1 month post-stroke was defined as an MMSE score ≤20 or a MoCA score <17 (31).

### Statistical analysis

Homocysteine levels were analyzed both as a continuous variable and as a categorical variable. Given the skewed distribution of serum homocysteine levels, a natural logarithmic transformation was applied prior to analysis. For normally distributed continuous data, mean ± SD values were used to describe the data, and independent-sample t-tests were employed to assess group differences. For skewed distributions, median values with interquartile ranges (IQR) were reported, and the Mann–Whitney U test was used to compare two groups. Categorical variables were presented as percentages, and differences between groups were assessed using the Chi-square test.

Multiple linear regression and logistic regression analyses were conducted to estimate the regression coefficients (β) and odds ratios (OR) for the association between homocysteine levels and post-stroke cognitive impairment. Patients were divided into two groups based on a cutoff of 15 μmol/L for homocysteine levels. Crude and multivariable-adjusted β and OR values with 95% confidence intervals (CIs) were calculated for cognitive scores and moderate-to-severe post-stroke cognitive impairment. These analyses were performed both categorically (using <15 μmol/L as the reference group) and continuously (per 1-unit increase in the homocysteine level, equivalent to a 2.7-fold increase).

Three models were constructed with progressively increased adjustments for potential confounding variables that could affect the association between homocysteine levels and cognitive function. The first model was Model 0 (unadjusted). Model 1 was adjusted for age, sex, BMI, education, history of diabetes and hypertension, stroke subtypes, NIHSS score, smoking and drinking status, systolic blood pressure, and serum levels of total cholesterol, triglycerides, vitamin B12, folate, and fasting glucose. Model 2 included further adjustment for eGFR. A two-tailed *p*-value of <0.05 was considered statistically significant. All analyses were performed using EmpowerStats software^1^ and the statistical package R.^2^

## Results

### Population characteristics

The clinical and demographic characteristics of all 608 patients, grouped by homocysteine levels, are presented in Table 1. The mean age of patients was 66.6 ± 4.1 years, and 48% were female. The median homocysteine level was 13.8 μmol/L (interquartile range [IQR]: 11.3–17.3 μmol/L). A total of 239 patients (39.3%) had total homocysteine levels higher than the cutoff of 15 μmol/L. Patients with higher homocysteine levels were more likely to be older, male, former or current drinkers, and smokers. They were also more likely to have high diastolic blood pressure, low folate and vitamin B12 levels, and low eGFR at admission, but less likely to have diabetes (Table 1).

**Table 1:** Demographic and clinical characteristics of patients stratified by serum homocysteine.

Variables	Overall	Homocysteine (μmol/L)		*p* value
		<15	≥15	
	*n* = 608	*n* = 369	*n* = 239	
Age, y (mean ± SD)	66.6 ± 4.1	66.2 ± 4.1	67.2 ± 4.2	0.002
Male, *n* (%)	316 (52.0)	154 (41.7)	162 (67.8)	<0.001
BMI,^a^ kg/m^2^ (mean ± SD)	25.0 ± 3.6	25.0 ± 3.4	24.8 ± 3.8	0.430
Education (> 6 years), *n* (%)	200 (32.9)	104 (28.2)	96 (40.2)	0.002
Systolic blood pressure on admission, mmHg (mean ± SD)	171.9 ± 20.2	171.5 ± 19.6	172.5 ± 21.1	0.816
Diastolic blood pressure on admission, mmHg (mean ± SD)	92.9 ± 12.2	91.7 ± 12.3	94.8 ± 11.9	<0.001
Smoking, *n* (%)				<0.001
Never	355 (58.5)	250 (67.9)	105 (43.9)	
Former	64 (10.5)	35 (9.5)	29 (12.1)	
Current	188 (31.0)	83 (22.6)	105 (43.9)	
Alcohol drinking, *n* (%)				0.003
Never	389 (64.0)	256 (69.4)	133 (55.6)	
Former	52 (8.6)	27 (7.3)	25 (10.5)	
Current	167 (27.5)	86 (23.3)	81 (33.9)	
Stroke subtypes, *n* (%)				0.640
Large artery	281 (46.2)	170 (46.1)	111 (46.4)	
Small vessel occlusion	309 (50.8)	189 (51.2)	120 (50.2)	
Cardioembolism	13 (2.1)	6 (1.6)	7 (2.9)	
Other determined etiology	3 (0.5)	2 (0.5)	1 (0.4)	
Undetermined etiology	2 (0.3)	2 (0.5)	0 (0.0)	
Diabetes mellitus,^b^ *n* (%)	50 (8.2)	38 (10.3)	12 (5.0)	0.021
Hypertension, *n* (%)	361 (59.4)	220 (59.6)	141 (59.0)	0.878
Laboratory results				
Total cholesterol, mmol/L (mean ± SD)	5.7 ± 1.2	5.7 ± 1.2	5.8 ± 1.2	0.454
Triglyceride, mmol/L (median, IQR)	1.4 (1.0–2.0)	1.4 (1.0–1.9)	1.4 (1.0–2.0)	0.579
Folate, ng/mL (mean ± SD)	7.5 ± 3.3	8.1 ± 3.2	6.5 ± 3.3	<0.001
Vitamin B12, pmol/L (mean ± SD)	399.5 ± 165.5	429.6 ± 178.7	353.4 ± 130.3	<0.001
Hcy, μmol/L (median, IQR)	13.8 (11.3–17.3)	11.7 (10.5–13.4)	18.3 (16.4–24.7)	<0.001
Fasting glucose, mmol/L (mean ± SD)	6.1 ± 1.8	6.2 ± 1.9	6.0 ± 1.6	0.295
Estimated glomerular filtration rate, ml/min/1.73m^2^ (median, IQR)	91.7 (18.5–148.1)	92.7 (88.3–96.4)	89.5 (73.1–94.5)	<0.001
Neural function assessment, mean (SD)				
MMSE score at 1 month, mean ± SD (range 0–30)	21.4 ± 2.1	21.6 ± 1.8	21.2 ± 2.5	0.129
MoCA score at 1 month, mean ± SD (range 0–30)	17.9 ± 2.3	18.2 ± 1.9	17.6 ± 2.8	0.029
NIHSS score at admission, mean ± SD (range 0–42)	5.0 ± 2.2	5.0 ± 2.2	5.1 ± 2.3	0.576

Hcy, Homocysteine; BMI, body mass index; MMSE, Mini-Mental State Examination; MoCA, Montreal Cognitive Assessment; NIHSS, National Institutes of Health Stroke Scale. Values are presented as mean ±SD or median (interquartile range, IQR) for continuous variables and *n* (%) for categorical variables.

a BMI was calculated as weight in kilograms divided by height in meters squared.

b Diabetes mellitus was defined as self-reported physician diagnosed diabetes.

c Hypertension was defined as self-reported physician diagnosed hypertension.

### Homocysteine levels and post-stroke cognitive impairment

Table 2 presents the results of analyses with unadjusted and adjusted models for continuous homocysteine levels and categorized homocysteine levels in relation to post-stroke cognitive impairment. When examined as a continuous variable (per 1-unit increase) in both the Model 0 and Model 1, the log-transformed increase in homocysteine level was significantly and negatively associated with MMSE and MoCA scores (for MMSE: β = −0.69, 95% CI: −1.16, −0.22, *p* = 0.004; for MoCA: β = −0.86, 95% CI: −1.38, −0.34, *p* = 0.001 in Model 1). However, after the inclusion of eGFR in Model 2, the association between the log-transformed homocysteine level and post-stroke cognitive impairment was no longer significant (for MMSE: β = 0.26, 95% CI: −0.18, 0.69, *p* = 0.253; for MoCA: β = 0.42, 95% CI: −0.04, 0.87, *p* = 0.073 in Model 2).

**Table 2:** Linear regression analysis of the association between serum homocysteine levels and post-stroke cognitive measures.

Hcy level (μmol/L)	No. of patients (%)	Model 0		Model 1		Model 2	
		ß (95%CI)	*p*	ß (95%CI)	*p*	ß (95%CI)	*p*
MMSE							
<15	369 (60.7)	0		0		0	
≥15	239 (39.3)	−0.44 (−0.78, −0.10)	0.011	−0.42 (−0.78, −0.06)	0.024	0.23 (−0.10, 0.57)	0.177
Log Hcy per 1-unit increase	608 (100)	−0.57 (−0.99, −0.15)	0.008	−0.69 (−1.16, −0.22)	0.004	0.26 (−0.18, 0.69)	0.253
MoCA							
<15	369 (60.7)	0		0		0	
≥15	239 (39.3)	−0.62 (−1.00, −0.25)	0.001	−0.53 (−0.94, −0.13)	0.010	0.34 (−0.01, 0.69)	0.056
Log Hcy per 1-unit increase	608 (100)	−0.85 (−1.31, −0.38)	<0.001	−0.86 (−1.38, −0.34)	0.001	0.42 (−0.04, 0.87)	0.073

In categorical analysis, a significant association was observed for patients with homocysteine levels ≥15 μmol/L compared with those with homocysteine levels <15 μmol/L in the Model 0 and Model 1 (for MMSE: β = −0.44, 95% CI: −0.78, −0.10, *p* = 0.011 in the Model 0; β = −0.42, 95% CI: −0.78, −0.06, *p* = 0.024 in Model 1; for MoCA: β = −0.62, 95% CI: −1.00, −0.25, *p* = 0.001 in the Model 0; β = −0.53, 95% CI: −0.94, −0.13, *p* = 0.010 in Model 1). Similarly, the direction of the relationship between homocysteine levels and post-stroke cognitive impairment changed once eGFR was introduced into Model 2 (for MMSE: β = 0.23, 95% CI: −0.10, 0.57, *p* = 0.177; for MoCA: β = 0.34, 95% CI: −0.04, 0.87, *p* = 0.056 in Model 2).

Table 3 presents the incidence of moderate-to-severe cognitive impairment 1 month after stroke, stratified by different serum homocysteine levels. A total of 68 (56.7%) and 96 (42.7%) patients exhibited moderate-to-severe post-stroke cognitive impairment, based on MoCA and MMSE scores, respectively. In both the Model 0 model and Model 1, compared with patients in the homocysteine level < 15 μmol/L group, those with homocysteine levels ≥ 15 μmol/L had a higher risk of moderate-to-severe post-stroke cognitive impairment (unadjusted OR = 2.42, 95% CI: 1.62, 3.64; *p* < 0.001 for MoCA in Model 0; adjusted OR = 2.95, 95% CI: 1.81, 4.79; *p* < 0.001 for MoCA in Model 1). However, this association was not significant once eGFR was introduced into the model (OR = 1.19, 95% CI: 0.66, 2.15; *p* = 0.557 for MoCA in Model 2). Similar results were observed when homocysteine level was analyzed as a continuous variable. The log-transformed homocysteine level was not significantly associated with the risk of moderate-to-severe post-stroke cognitive impairment for MoCA scores in Model 2 (*p* = 0.955).

**Table 3:** Logistic regression analysis of the association between Hcy levels and moderate to severe post-stroke cognitive impairment.

Hcy level (μmol/L)	No. of patients (%)	No. of PSCI (%)	Model 0		Model 1		Model 2	
			OR (95%CI)	*p*	OR (95%CI)	*p*	OR (95%CI)	*p*
MMSE								
<15	369 (60.7)	129 (35.0)	1		1		1	
≥15	239 (39.3)	96 (40.2)	1.25 (0.89, 1.75)	0.194	1.28 (0.86, 1.89)	0.226	0.82 (0.53, 1.27)	0.247
Log Hcy per 1-unit increase	608 (100)	225 (37.0)	1.42 (0.94, 2.14)	0.097	1.65 (0.99, 2.75)	0.056	0.88 (0.49, 1.57)	0.668
MoCA								
<15	369 (60.7)	52 (14.1)	1		1		1	
≥15	239 (39.3)	68 (28.5)	2.42 (1.62, 3.64)	<0.001	2.95 (1.81, 4.79)	<0.001	1.19 (0.66, 2.15)	0.557
Log Hcy per 1-unit increase	608 (100)	120 (19.7)	2.57 (1.61, 4.11)	<0.001	3.55 (1.97, 6.42)	<0.001	0.98 (0.45, 2.11)	0.955

### Stratified analyses by potential effect modifiers

The relationship between homocysteine levels (≥15 μmol/L vs. <15 μmol/L) and post-stroke cognitive performance was further evaluated using stratified analysis (Table 4). A fully multivariable-adjusted significant negative association between elevated homocysteine levels and post-stroke cognitive performance was observed in patients with eGFR < 90 ml/min/1.73 m^2^, with significant interactions between eGFR and MMSE scores (*P* for interaction = 0.005) and MoCA scores (*P* for interaction = 0.001). In contrast, other variables, including sex, age, NIHSS score, systolic blood pressure, stroke subtypes, and blood glucose levels, did not significantly mediate the relationship between homocysteine levels and post-stroke cognitive impairment.

**Table 4:** Multivariable adjusted* linear regression of MMSE/MoCA scores with serum homocysteine within subgroups.

Subgroups	MMSE		*P* for interaction	MoCA		*P* for interaction
	<15 μmol/L	≥15 μmol/L		<15 μmol/L	≥15 μmol/L	
	ß (95%CI) *P*			ß (95%CI) *P*		
Gender			0.384			0.175
Male	0	0.45 (0.01, 0.89) 0.046		0	0.62 (0.17, 1.07) 0.007	
Female	0	−0.09 (−0.65, 0.47) 0.751		0	−0.09 (−0.68, 0.50) 0.762	
Age dichotomous			0.149			0.807
Lower<67 years	0	0.09 (−0.42, 0.59) 0.739		0	0.24 (−0.31, 0.79) 0.392	
Higher≥67 years	0	0.31 (−0.16, 0.78) 0.202		0	0.44 (−0.03, 0.90) 0.065	
Systolic blood pressrue dichotomous			0.750			0.653
Lower<171.9 mmHg	0	0.18 (−0.31, 0.68) 0.468		0	0.32 (−0.20, 0.83) 0.228	
Higher≥171.9 mmHg	0	0.29 (−0.18, 0.76) 0.224		0	0.37 (−0.12, 0.86) 0.142	
NIHSS score dichtomous			0.681			0.550
Lower<5	0	0.31 (−0.21, 0.84) 0.242		0	0.47 (−0.07, 1.01) 0.088	
Higher≥5	0	0.26 (−0.19, 0.71) 0.263		0	0.27 (−0.20, 0.75) 0.258	
Stroke subtypes			0.450			0.804
Large artery	0	0.21 (−0.33, 0.75) 0.443		0	0.26 (−0.29, 0.82) 0.348	
Small vessel occlusion	0	0.26 (−0.19, 0.70) 0.266		0	0.40 (−0.08, 0.88) 0.102	
eGFR categoriess			0.005			0.001
<90 ml/min/1.73m^2^	0	0.24 (−0.22, 0.70) 0.301		0	0.41 (−0.07, 0.90) 0.094	
≥90 ml/min/1.73m^2^	0	−0.72 (−1.35, −0.08) 0.028		0	−0.88 (−1.58, −0.19) 0.013	
Blood glucose dichotomous			0.692			0.788
Lower <6.1 mmol/L	0	0.17 (−0.29, 0.63) 0.472		0	0.31 (−0.19, 0.80) 0.231	
Higher ≥6.1 mmol/L	0	0.32 (−0.18, 0.82) 0.206		0	0.45 (−0.05, 0.96) 0.082	

### Joint effects of homocysteine and eGFR levels on post-stroke cognitive impairment

Based on the data provided in Table 5, the joint analysis of serum homocysteine and eGFR levels on moderate to severe post-stroke cognitive impairment revealed significant insights into the risk factors associated with PSCI. The logistic regression analyses revealed that the highest risk of PSCI was in the group with eGFR < 90 ml/min/1.73 m^2^ and homocysteine levels ≥15 μmol/L. After adjusting for pertinent confounders, the odds ratios (ORs) were 2.50 (95% CI: 1.49, 4.18; *p* < 0.001) for MMSE and 13.53 (95% CI: 6.64, 27.56; *p* < 0.001) for MoCA.

**Table 5:** Joint analysis of the serum homocysteine (<15 vs. ≥15) and eGFR (<90 vs. ≥90) levels on moderate to severe post-stroke cognitive impairment.

eGFR level (ml/min/1.73m^2^)	Hcy level (μmol/L)	No. of patients (%)	No. of PSCI (%)	Unadjusted		Multivariable adjusted^*^	
				OR (95%CI)	*p*	OR (95%CI)	*p*
MMSE							
≥90	<15	256 (42.1)	74 (28.9)	1		1	
	≥15	115 (18.9)	30 (26.1)	0.87 (0.53, 1.43)	0.576	1.00 (0.58, 1.75)	0.986
<90	<15	113 (18.6)	55 (48.7)	2.33 (1.48, 3.68)	<0.001	2.16 (1.30, 3.61)	0.003
	≥15	124 (20.4)	66 (53.2)	2.80 (1.79, 4.36)	<0.001	2.50 (1.49, 4.18)	<0.001
MoCA							
≥90	<15	256 (42.1)	21 (8.2)	1		1	
	≥15	115 (18.9)	11 (9.6)	1.18 (0.55, 2.54)	0.666	1.68 (0.71, 3.96)	0.239
<90	<15	113 (18.6)	31 (27.4)	4.23 (2.30, 7.77)	<0.001	5.38 (2.64, 10.95)	<0.001
	≥15	124 (20.4)	57 (46.0)	9.52 (5.39, 16.82)	<0.001	13.53 (6.64, 27.56)	<0.001

ß: standardized regression coefficient; CI: confidence interval; Hcy, Homocysteine; eGFR: estimated glomerular filtration rate; NIHSS: National Institutes of Health Stroke Scale. PSCI: post-stroke cognitive impairment.

* Adjusted for age, gender, BMI, systolic blood pressure, education, diabetes and hypertension history, stroke subtypes, NIHSS scores, smoking and alcohol drinking status, serum total cholesterol, triglyceride, vitamin B12, folate and fasting glucose.

## Discussion

This study highlight the complex interplay between homocysteine levels and kidney function, as indicated by eGFR, on cognitive impairment post-stroke. Higher levels of homocysteine and reduced eGFR are significantly associated with an increased risk of PSCI, as observed in both the MMSE and MoCA assessments. These findings underscore the importance of managing plasma homocysteine levels and monitoring kidney function in stroke patients to mitigate the risk of cognitive decline (25, 32, 33).

To date, considerable data on homocysteine levels and PSCI have been published. However, the available data are controversial (34, 35) and the relationship with renal function remains to be elucidated. A systematic review study found a positive association between cognitive decline and elevated plasma homocysteine levels in the general population and patients with cognitive dysfunction. However, treatment with vitamin supplementation failed to show improved cognitive decline (14). A recent Chinese cohort of minor stroke/TIA patients (NIHSS 1.49 ± 1.31) reported that elevated baseline homocysteine (≥ 15 μmol/L) predicted PSCI at 12 months, but not at 3 months (MoCA < 22), in women (35). In contrast, our patients exhibited more severe neurological deficits (NIHSS 5.0 ± 2.2) and pronounced early cognitive impairment (MoCA < 17; MMSE < 20 at 1 month). Similarly, another Chinese study demonstrated that acute-phase hyperhomocysteinaemia (≥ 12 μmol/L) was independently associated with PSCI at 1 month in patients with baseline NIHSS 1 (IQR 0–3) and MMSE < 24 as the diagnostic threshold (22). Collectively, the discrepant findings across studies can be attributed to differences in initial stroke severity, cognitive assessment tools and diagnostic criteria, and the interval between stroke onset and evaluation. One study found that higher serum homocysteine levels and an increased vascular burden were negative related to executive function in patients with CKD (36). The Randomized FAVORIT Assisted Cognitive Trial found cognitive benefit in kidney transplant recipients with elevated baseline homocysteine levels with high doses of vitamin B supplementation (32). Our study revealed that patients with elevated homocysteine levels and lower eGFR exhibited a significant association with PSCI. These findings clearly showed the additive value of hyperhomocysteinemia and lower eGFR in predicting incident PSCI risk.

The exact pathophysiologic mechanisms that underlie the link between homocysteine levels, eGFR, and PSCI are not fully established and require further elucidation. Hyperhomocysteinemia induces oxidative stress and antagonizes the vasodilator properties of NO though the formation of S-nitrosohomocysteine, leading to endothelial dysfunction (37). Similarly, hyperhomocysteinemia causes vascular hypertrophy and remodeling, impairs the basic characteristics of blood vessels, and increases the stiffness of arteries (38). Furthermore, homocysteine has been shown to promote the proliferation of smooth muscle cells, leading to interactions with platelets, clotting factors, and lipids (39). Chronic elevated serum homocysteine alters functions of the vascular endothelial cells, and based on important pathobiochemical modifications, activates thiolation and homocysteinylation of plasma proteins and enzymes with a deleterious impact on cerebrovascular permeability and eventually on brain parenchyma (15).

The association between eGFR and homocysteine levels has been documented in numerous studies (24). Evidence have consistently shown a highly significant negative correlation between eGFR and homocysteine levels. This provides compelling indirect evidence that elevated homocysteine levels in patients with renal disease are closely linked to impaired kidney function (23). In our cohort, higher homocysteine levels were associated with lower eGFR. After adjustment for eGFR (Model 2), the β-coefficients for log-transformed homocysteine shifted from negative in Model 1 (MMSE: β = −0.69; MoCA: β = −0.86) to positive (MMSE: β = 0.26; MoCA: β = 0.42), and the association between homocysteine and PSCI became non-significant. These observations suggest that eGFR may mediate the relationship, although the underlying pathophysiological mechanisms remain to be elucidated. Organs such as the kidney, liver, gut, and pancreas contain the enzymes needed for homocysteine metabolism. The kidney plays an important role in clearing homocysteine from circulation. Patients with CKD and those who are predisposed to high blood Hcy have a markedly lower serum Hcy clearance (40). Moreover, CKD heightens the risk of elevated blood Hcy levels, which has been linked to a decrease in cognition (41). In addition, it is important to emphasize that decreased GFR is a well-known risk factor for cognitive impairment (42). The coexistence of low eGFR and hyperhomocysteinemia may also contribute to the accumulation of uremic toxins owing to the deterioration of the renal clearance function, causing direct neurotoxicity, or be accompanied by systemic hemodynamic impairment, both of which lead to the development of vascular cognitive impairment (26, 43). The additional detrimental effects of these markers on brain function remain unclear, warranting further research.

The strengths of this study include the assessment of cognitive function with both MoCA and MMSE, thus providing clinical accuracy to the analyses. In addition, we were able to control for many potential confounders including demographic and clinical indicators to reduce confounding effects. However, there were several limitations in our study. First, the homocysteine level was measured once, and thus possible intra-individual fluctuations were ignored. Second, we did not analyze impairment in specific cognitive domains, but only compared overall cognitive domains. Third, we lacked data on diet or post-diagnosis B-vitamin supplementation—both of which can alter homocysteine levels and bias the observed association. These variables will be collected in subsequent follow-up. Finally, cognitive assessment was assessed 1 month after discharge, and the incidence of PSCI was the highest 3 months after stroke (44). Further long-term follow-up should be performed.

## Conclusion

Our findings indicates that the patients with hyperhomocysteinemia and low eGFR levels exhibited a significant association with PSCI. These results clearly showed the additive value of hyperhomocysteinemia and lower eGFR in predicting incident PSCI risk. These findings have important clinical implications. These findings underscore the importance of managing plasma homocysteine levels and monitoring kidney function in stroke patients to mitigate the risk of cognitive decline. Further investigation into the mechanisms behind elevated homocysteine and impaired kidney function could provide essential insights into prevention strategies for PSCI in post-stroke patients. Implementing interventions that target these modifiable risk factors could enhance cognitive outcomes following a stroke.

## Data Availability

The datasets presented in this study can be found in online repositories. The names of the repository/repositories and accession number(s) can be found in the article/Supplementary material.

## Footnotes

^1^ www.EmpowerStats.com

^2^ www.r-project.org
